# Residual Microcalcifications After Neoadjuvant Chemotherapy: Implications for Surgical Decision-Making—A Systematic Review

**DOI:** 10.3390/jcm15020451

**Published:** 2026-01-07

**Authors:** Yun Yeong Kim, Hyun Jik Kim, Yong Soon Chun, Heung Kyu Park

**Affiliations:** Department of General Surgery, Breast Cancer Center, Gachon University Gil Medical Center, Incheon 21565, Republic of Korea

**Keywords:** breast cancer, neoadjuvant chemotherapy, microcalcifications, pathologic complete response, MRI, breast-conserving surgery

## Abstract

**Background**: The clinical and oncologic significance of residual microcalcifications after neoadjuvant chemotherapy (NAC) in breast cancer remains poorly defined. While traditionally regarded as radiologic indicators of residual malignancy warranting complete surgical excision, accumulating evidence suggests that many post-treatment calcifications represent benign or in situ changes with limited prognostic relevance. This systematic review synthesizes current evidence to clarify the diagnostic, pathologic, and oncologic implications of persistent calcifications after NAC. **Methods**: Following PRISMA 2020 guidelines, we conducted a comprehensive search of PubMed, Embase, the Cochrane Library, Scopus, and Google Scholar for studies published between January 2000 and May 2025. Eligible studies included adult breast cancer patients treated with NAC who demonstrated residual calcifications on mammography or MRI with corresponding histopathologic or survival data. Two reviewers independently performed study selection, data extraction, and quality assessment using the Newcastle–Ottawa Scale and AMSTAR-2. **Results**: Twenty-four studies involving over 3000 patients were included. Across cohorts, 35–55% of residual calcifications were benign, and many others corresponded to ductal carcinoma in situ rather than invasive carcinoma. Calcifications frequently persisted despite pathologic complete response (pCR), particularly in HER2-positive and triple-negative subtypes. MRI demonstrated superior concordance with pathology compared with mammography. Persistent calcifications did not consistently correlate with worse disease-free or overall survival when pCR was achieved. Radiologic–pathologic discordance contributed to overtreatment in some cohorts, including unnecessary mastectomy or extensive resections. **Conclusions**: Residual calcifications after NAC should not be regarded as a definitive surrogate of residual invasive disease nor as an obligatory indication for complete surgical removal. Their frequent benign or in situ pathology and limited prognostic value support a more individualized approach to surgical planning, prioritizing pathologic response and margin status over radiographic calcifications alone.

## 1. Introduction

The introduction of neoadjuvant chemotherapy (NAC) has transformed the clinical approach to breast cancer management [1,2]. In addition to shrinking tumors and thereby expanding the feasibility of breast-conserving surgery, NAC offers a valuable window into tumor biology by allowing direct evaluation of treatment response in vivo. Pathologic complete response (pCR) has emerged as a strong surrogate endpoint for long-term survival, particularly in biologically aggressive yet chemo-sensitive subtypes such as HER2-positive and triple-negative breast cancers [3,4,5]. As a result, accurate evaluation of treatment response has become central to surgical planning and prognostic assessment. In the post-NAC setting, surgical decision-making is increasingly guided by treatment response rather than initial tumor extent; however, determining the appropriate extent of surgery remains challenging when residual imaging abnormalities persist despite apparent clinical or radiologic complete response.

One persistent challenge, however, is the interpretation of residual imaging abnormalities after NAC. Mammographic microcalcifications, in particular, have long been regarded as markers of residual malignancy. Because calcifications may correspond to invasive carcinoma or ductal carcinoma in situ (DCIS), the prevailing surgical doctrine has traditionally favored complete excision of calcified areas to maximize oncologic safety. Yet, this practice has been increasingly contested, as growing evidence indicates that a significant proportion of post-NAC calcifications represent benign, therapy-related changes—such as fibrosis, necrosis, or stromal remodeling—rather than viable tumor [6,7,8,9]. Moreover, residual calcifications are frequently observed even in patients who achieve pCR. Mammography remains the standard modality for evaluating microcalcifications, but its ability to distinguish viable tumor from therapy-related changes is limited. Although MRI more accurately reflects residual invasive disease, it may underestimate residual ductal carcinoma in situ, contributing to radiologic–pathologic discordance.

The prognostic relevance of these calcifications remains unsettled. While certain studies have associated their persistence with residual disease, others have found no adverse impact on disease-free or overall survival among patients who achieved pCR [10,11,12,13]. Radiologic–pathologic discordance appears particularly pronounced in HER2-positive and triple-negative cancers, where calcifications often remain despite high rates of pCR. This raise concerns that calcification-directed surgical strategies may lead to overtreatment—such as unnecessary mastectomy or extensive resections—without yielding meaningful survival benefit [12,13,14,15]. Consequently, current guidelines and clinical practice remain inconsistent, and no clear consensus exists regarding whether all residual calcifications after NAC require complete surgical excision, particularly in patients who achieve pCR.

In light of these uncertainties, we conducted a systematic review to critically appraise the clinical and oncologic significance of residual microcalcifications after NAC. The primary objective was to determine whether routine surgical excision of such calcifications is warranted, or whether their persistence can be safely observed in patients who achieve pCR. By synthesizing evidence across imaging, pathology, and survival outcomes, this review seeks to inform surgical decision-making and clarify the role of calcifications in guiding operative extent in contemporary, individualized breast cancer therapy.

Systematic reviews specifically addressing the clinical significance of residual microcalcifications after neoadjuvant chemotherapy are scarce. Existing reviews have largely focused on imaging performance or diagnostic accuracy, with limited attention to surgical decision-making or long-term oncologic outcomes. In this context, the present systematic review provides a comprehensive synthesis of imaging, pathology, and survival data to clarify the clinical implications of persistent calcifications and to inform contemporary strategies for surgical planning and treatment de-escalation after NAC.

## 2. Methods

### 2.1. Search Strategy and Study Selection

**Figure 1:**
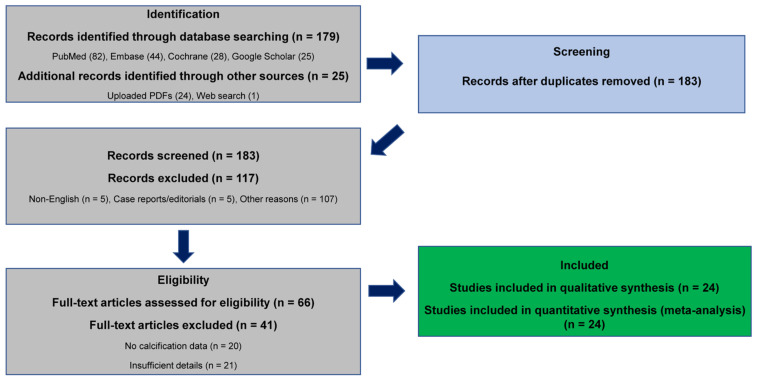
PRISMA flow diagram showing the process of study identification, screening, eligibility assessment, and inclusion in the systematic review.

This systematic review adhered to the PRISMA 2020 guidelines for transparent reporting of evidence synthesis [16]. Because the study synthesized previously published data without individual patient-level information, the protocol was not prospectively registered in PROSPERO. Comprehensive electronic searches were undertaken in PubMed, Embase, the Cochrane Library, Scopus, and Google Scholar for studies published between January 2000 and May 2025, using both controlled vocabulary (MeSH and Emtree terms) and relevant free-text keywords including “breast cancer,” “microcalcifications,” and “neoadjuvant chemotherapy.” Detailed, reproducible search strategies for each database are presented in Supplementary Table S2. Study selection followed predefined inclusion and exclusion criteria, and the overall process is summarized in the PRISMA flow diagram (Figure 1). A completed PRISMA 2020 checklist is provided as Supplementary Material.

Two reviewers independently screened titles, abstracts, and full texts, documenting reasons for exclusion. Disagreements were resolved by consensus or by a third reviewer when necessary. Inter-rater reliability was assessed using Cohen’s kappa (κ), with values > 0.60 considered substantial.

### 2.2. Data Extraction and Quality Assessment

Data were extracted into a standardized form, capturing study characteristics, patient and tumor profiles, imaging modalities, calcification patterns, histopathology, and survival outcomes. Risk of bias was assessed using the Newcastle–Ottawa Scale (NOS) for cohort and case–control studies, and AMSTAR-2 for systematic reviews [17]. Studies were categorized as low (7–9), moderate (4–6), or high (0–3) risk of bias. Due to heterogeneity in study design, NAC regimens, and outcome definitions, quantitative meta-analysis was not feasible. Therefore, results were synthesized qualitatively, emphasizing descriptive pooling of calcification pathology (benign vs. malignant; DCIS vs. invasive) and comparative performance between mammography and MRI.

Reporting biases were not formally assessed because the included studies were highly heterogeneous.

### 2.3. Study Inclusion

A total of 204 records were identified across databases (PubMed = 82, Embase = 44, Cochrane = 28, Google Scholar = 25, and 25 from hand-searching), of which 21 duplicates were removed, leaving 183 unique records for screening. Following title/abstract and full-text review, 24 studies met the eligibility criteria and were included in the final synthesis. Key study characteristics including design, sample size, NAC regimen, imaging modality, and pathologic correlation (% benign, % DCIS, % invasive) were summarized in Supplementary Table S1 for clarity and transparency. (Figure 1; Supplementary Table S1).

### 2.4. Data Synthesis and Study Heterogeneity

Owing to heterogeneity in study designs, NAC regimens, and outcome definitions, no quantitative meta-analysis was performed. Instead, findings were qualitatively synthesized, with descriptive pooling of calcification patterns (benign vs. malignant, DCIS vs. invasive) and comparative performance of mammography versus MRI. To further illustrate inter-study variability, a summary of representative studies highlighting heterogeneity in NAC regimens, imaging modalities, calcification classification, and pCR definitions was provided in Supplementary Table S3.

### 2.5. Risk of Bias

#### 2.5.1. Study Design-Related Bias

Most included studies were retrospective in design, which introduces inherent risks of selection bias and unmeasured confounding. Prospective cohort studies were methodologically stronger but were limited in number and often underpowered, restricting causal inference. Pathology review and clinical follow-up were generally well documented across studies.

#### 2.5.2. Imaging Heterogeneity and Outcome Definition

Considerable heterogeneity existed in imaging protocols and response assessment across studies, including differences in the use of mammography, MRI, and contrast-enhanced mammography. In addition, definitions of pathologic complete response varied, particularly regarding the handling of residual ductal carcinoma in situ, which may have contributed to variability in reported outcomes.

#### 2.5.3. Reporting Quality and Follow-Up Limitations

Reporting of oncologic outcomes was inconsistent across studies, with variable follow-up durations and incomplete reporting of disease-free or overall survival in several cohorts, limiting robust comparative interpretation. The two included systematic reviews were of moderate methodological quality based on AMSTAR-2 assessment and neither had preregistered protocols [17].

#### 2.5.4. Supplementary Analyses and Supporting Evidence

Additional supporting evidence and methodological details from previous studies are provided in the Supplementary Materials [18,19,20,21,22,23,24,25,26].

## 3. Results

### 3.1. Study Characteristics

The eligible studies, published between 2010 and 2025, comprised predominantly retrospective cohorts (*n* = 18), with two prospective observational cohorts and three systematic reviews. Sample sizes ranged from 40 to more than 380 patients, with meta-analyses including over 5000 participants. Most investigations assessed anthracycline- and taxane-based NAC regimens, with HER2-positive subgroups frequently receiving trastuzumab with or without pertuzumab. Mammography (MG) was used in all studies, while MRI was included in 12 and contrast-enhanced mammography (CEM) in 2. Reported outcomes focused on pathologic complete response (pCR), residual disease (DCIS or invasive carcinoma), and survival endpoints such as disease-free survival (DFS) and overall survival (OS).

### 3.2. Imaging–Pathology Correlation

MRI consistently outperformed MG in estimating residual tumor burden. Zhu et al. reported an intraclass correlation coefficient (ICC) of 0.77 between MRI and pathology compared with 0.10 for MG, while An et al. demonstrated concordance correlation coefficients of 0.566 for MRI and 0.196 for MG, with greatest accuracy in ER-negative tumors [27,28]. Calcification dynamics were variable: Li et al. described decreases (40%), increases (7.5%), and stability (52.5%), none of which reliably predicted pCR [10]. In the prospective multicenter NRG-BR005 trial, Basik et al. evaluated 101 patients with clinical and radiologic complete response after NAC Using combined imaging (mammography, ultrasound, MRI) and stereotactic tumor-bed biopsy, the overall negative predictive value (NPV) was 78.3% (95% CI, 67.9–86.6%), below the prespecified 90% threshold [29]. In the HER2-positive subgroup, however, the NPV reached 90% (95% CI, 76.3–97.2%).

### 3.3. Pathologic Complete Response and Calcifications

Across cohorts, approximately 35–55% of post-treatment calcifications corresponded to benign therapy-related changes (fibrosis, necrosis, or stromal alterations), while the remainder were most often DCIS rather than invasive carcinoma. Multiple studies (Adrada et al., Goldberg et al., Ploumen et al.) consistently demonstrated that the persistence of calcifications after NAC does not reliably indicate residual invasive disease, supporting the limited oncologic value of calcification-driven resections [6,7,8].

### 3.4. Subtype-Specific Patterns of Residual Microcalcifications

Persistent calcifications were documented even in patients who achieved pCR. For example, Adrada et al. reported that among pCR cases, 66% of residual calcifications were benign and 28% were DCIS only, with no invasive component [7]. In aggressive but chemo-sensitive subtypes such as HER2-positive and triple-negative cancers, calcifications frequently persisted despite high pCR rates. Conversely, in hormone receptor–positive cancers, residual calcifications were more often associated with DCIS rather than invasive carcinoma.

### 3.5. Prognostic Outcomes

The presence of residual calcifications did not consistently influence prognosis when pCR was achieved. Patients with residual invasive disease had worse outcomes, but this was independent of calcification status. In the largest cohort, Kim et al. (*n* = 381) demonstrated that the presence of residual calcifications did not significantly affect DFS or OS in patients achieving pCR [11]. Local recurrence was rare when negative margins were obtained, regardless of whether residual calcifications were resected. In a more recent series, Allotey et al. evaluated 42 patients and reported no significant association between residual calcifications and pCR (*p* = 0.763), nor with recurrence (HR 2.599; 95% CI: 0.290–23.264; *p* = 0.393) or overall survival (HR 1.362; 95% CI: 0.123–15.062; *p* = 0.801), with Kaplan–Meier analysis further confirming no survival differences according to calcification status [12]. Reporting bias could not be formally evaluated because no meta-analysis was performed.

## 4. Discussion

### 4.1. Historical Perspective and Radiologic–Pathologic Discordance After NAC

Residual mammographic microcalcifications after neoadjuvant chemotherapy (NAC) have traditionally been viewed as signs that some disease may remain. This idea came from early reports in which persistent calcifications were often associated with ductal carcinoma in situ (DCIS) and, in a smaller number of cases, invasive cancer. Because of this background, surgeons have generally preferred to remove all calcified areas to avoid leaving residual disease and to reduce the chance of local recurrence.

Much of this practice also reflected the limits of radiologic–pathologic agreement in earlier eras. Imaging could not always predict whether a calcified area truly represented active disease, so a more extensive surgical approach often felt safer. More recent studies, however, tell a somewhat different story. Several prospective and multicenter evaluations have shown that modern image-guided biopsies can confirm pathologic complete response (pCR) with a high level of reliability. Some groups have even explored the possibility of avoiding surgery altogether in very selected patients. These results force us to reconsider whether calcifications alone should continue to dictate the extent of resection [30,31].

### 4.2. Pathologic Significance of Residual Microcalcifications

Recent reports have begun to weaken the long-held assumption that persistent calcifications reliably signal remaining malignancy. Several retrospective studies have shown that many post-NAC calcifications are not related to viable tumor at all but instead reflect treatment-related changes in the stroma, including fibrosis, necrosis, and dystrophic calcification. A proportion still represents DCIS and therefore requires complete excision, but a large number arise from benign post-therapeutic processes. This difference matters in practice, because only the latter group reasonably supports a more conservative surgical plan.

Adrada et al. [7] and Goldberg et al. [6] found that roughly 40% to more than 70% of calcifications after NAC were benign or nonviable. An et al. [27] reported that persistent calcifications were more often associated with DCIS than with invasive disease. MRI has also shown better alignment with final pathology than mammography in several series, including the work by Zhu et al. [28]. A more recent synthesis by Ploumen et al. reached similar conclusions, noting that calcifications corresponded to DCIS in 29–60% of cases and to benign pathology in 38–62%. Their review, however, focused mainly on diagnostic accuracy. In contrast, our analysis incorporates available survival and recurrence outcomes, which reinforces the point that leaving behind benign calcifications does not appear to compromise oncologic safety.

### 4.3. Imaging Modality–Specific Considerations

From a radiologic perspective, persistent calcifications after NAC can complicate both surveillance and interpretation. Benign calcifications may resemble recurrent disease on mammography, leading to additional biopsies or closer follow-up. Interpretation becomes even more challenging in dense breasts or in the presence of post-treatment architectural distortion, and inter-observer variability remains considerable. These issues highlight the need for more consistent imaging criteria to help distinguish benign post-therapy changes from true recurrence. Combining pre-NAC imaging with post-surgical pathology may improve accuracy in difficult cases.

### 4.4. Prognostic Implications of Residual Microcalcifications

Across multiple studies, pCR—not calcification status—emerges as the main driver of long-term outcomes. Investigations by Kim et al., Li et al., and Allotey et al. consistently show that persistent calcifications do not worsen disease-free or overall survival in patients who reach pCR. Changes in calcification patterns, whether decreasing, stable, or increasing, also fail to predict prognosis, which limits their usefulness as markers of survival [10,11,12].

### 4.5. Subtype-Specific and Biologic Considerations

Some investigators still take a more cautious stance. In a subset of patients, calcifications may continue to contain residual disease, and this seems to depend partly on tumor biology. Weiner et al. noted that changes in calcifications after NAC can reflect a response in the invasive component but do not necessarily indicate clearance of DCIS [32]. Talec et al. also pointed out that removing calcified areas may remain appropriate in hormone receptor–positive or multifocal disease, where the chance of finding residual carcinoma is still meaningful [33]. These views reflect the ongoing tension between enthusiasm for surgical de-escalation and the need to maintain oncologic safety.

Molecular subtype provides additional context. In HER2-positive and triple-negative cancers—subtypes known for aggressive behavior yet strong chemotherapy response—calcifications often remain despite high pCR rates. This creates frequent mismatch between imaging and pathology. In contrast, hormone receptor–positive disease tends to respond less dramatically to chemotherapy, and persistent calcifications in these tumors more commonly represent DCIS rather than benign effects of treatment. For this reason, subtype-specific interpretation is important. A more conservative resection may still be reasonable in HR^+^ tumors, while a more flexible approach may be acceptable in HER2-positive or triple-negative disease when pCR and margin clearance have been confirmed.

### 4.6. Implications for Surgical Decision-Making and Treatment De-Escalation

Considering these findings, the available evidence suggests that surgery guided strictly by the presence of calcifications may lead to more extensive operations than necessary, including occasional mastectomy, without a clear oncologic advantage. The prospective NRG-BR005 trial (Basik et al.) tested whether surgery could be omitted after NAC using MRI and stereotactic biopsy of the tumor bed [29]. MRI’s strong agreement with pathologic response supports its role as a key imaging tool in de-escalation strategies, especially for HER2-positive and triple-negative cancers. The study showed a negative predictive value of 78.3%, rising to 90% in HER2-positive tumors, reinforcing the importance of subtype-based selection. Importantly, the trial excluded patients with malignant calcifications, reflecting the continued use of calcifications as a safety factor in non-operative management.

In clinical practice, these findings suggest that surgical decisions after NAC should be guided primarily by pathologic response and margin status rather than the presence of calcifications alone. Baseline tumor burden and multifocality remain important determinants of surgical extent, even among patients who achieve pCR, and breast-conserving surgery continues to produce outcomes comparable to mastectomy [14]. Multidisciplinary interpretation—integrating imaging, pathology, and tumor biology—will be central to tailoring operative plans. In highly chemo-sensitive subtypes, benign calcifications confirmed by biopsy may not require wider excision when margins are clear and pCR is established. In contrast, more cautious surgery remains appropriate in HR^+^ tumors, where persistent calcifications are more often linked to DCIS.

**Figure 2:**
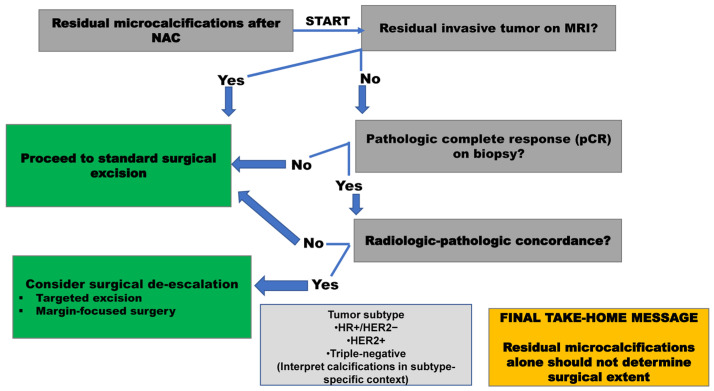
Clinical decision framework for interpreting residual microcalcifications after neoadjuvant chemotherapy. This algorithm illustrates a structured approach to surgical decision-making in patients with persistent microcalcifications following neoadjuvant chemotherapy. Residual invasive tumor on MRI, biopsy-proven pathologic complete response, and radiologic–pathologic concordance represent key decision points. Tumor subtype acts as a contextual modifier influencing interpretation at each stage. Importantly, residual microcalcifications alone should not be used as a surrogate marker of residual invasive disease nor as the sole determinant of surgical extent.

To translate these insights into clinical practice, we propose a clinical decision framework (Figure 2) that incorporates imaging features, biopsy confirmation, and molecular subtype to guide individualized surgical planning.

### 4.7. Limitations

This review has several limitations. First, most included studies were retrospective in design, which introduces inherent selection bias and limits causal inference. Second, there was substantial heterogeneity in neoadjuvant chemotherapy regimens and imaging protocols across studies, reducing comparability and precluding quantitative meta-analysis. Third, reporting of survival outcomes was inconsistent, with variable follow-up durations and endpoints across studies. Finally, definitions of residual microcalcifications and pathologic complete response were not standardized, reflecting the lack of uniform criteria in the existing literature. Despite these limitations, the overall findings of this review consistently support a more individualized approach to surgical decision-making after neoadjuvant chemotherapy.

## 5. Conclusions

Overall, the evidence points to a middle ground. Calcifications should not automatically be viewed as malignant or dismissed as irrelevant; their meaning depends on context. pCR and margin status remain the strongest guides for management, while calcifications serve as additional information whose significance varies by subtype and radiologic–pathologic alignment. Future research that combines advanced imaging, subtype-specific analysis, and biopsy confirmation will be important for refining patient selection and advancing safe de-escalation approaches after NAC. Understanding that many post-treatment calcifications are benign or in situ may allow surgeons to consider breast-conserving or omission strategies without compromising oncologic safety.

## Data Availability

No new datasets were generated for this study. All data used for analysis were derived from previously published articles and are fully documented within the manuscript and its Supplementary Materials.

## Supplementary Materials

The following supporting information can be downloaded at: https://www.mdpi.com/article/10.3390/jcm15020451/s1, Table S1: Summary of included studies; Table S2: Search strategies; Table S3: Study heterogeneity analysis.
