# Supplementary material for: Residual Microcalcifications After Neoadjuvant Chemotherapy: Implications for Surgical Decision-Making—A Systematic Review
Source: J Clin Med. 2026 Jan 7;15(2):451. doi: 10.3390/jcm15020451 (PMC12842292; doi:10.3390/jcm15020451)
Supplement: Supplementary file 1 [file jcm-15-00451-s001.zip › PRISMA-S_Checklist_MDPI.pdf]

PRISMA-S Checklist

1. Database name(s)

PubMed, Embase, Cochrane Library, Scopus, Google Scholar

2. Multi-database searching

Not applicable

3. Study registries

None — No study registries were searched.

4. Online resources and browsing

None

5. Citation searching

Reference lists of included articles manually screened

6. Search filters

No filters used except date limits (2000–2025)

7. Search updates

Not applicable

8. Dates of searches

Initial search: May 2025; No updates performed

9. Search strategy

Full electronic search strategy provided in Supplementary Table S2

10. Limitations

English language only
