# Supplementary material for: Residual Microcalcifications After Neoadjuvant Chemotherapy: Implications for Surgical Decision-Making—A Systematic Review
Source: J Clin Med. 2026 Jan 7;15(2):451. doi: 10.3390/jcm15020451 (PMC12842292; doi:10.3390/jcm15020451)
Supplement: Supplementary file 1 [file jcm-15-00451-s001.zip › PRISMA_2020_Checklist_.pdf]

PRISMA 2020 Checklist

Item	Checklist Item	Location
1 Title	Identify the report as a systematic review.	p.1
2 Abstract	See the PRISMA 2020 for Abstracts checklist.	p.2
3 Rationale	Describe the rationale for the review.	p.3
4 Objectives	State objectives/questions.	p.4-5
5 Eligibility criteria	Specify inclusion/exclusion criteria.	p.4-6
6 Information sources	All databases, dates searched.	p.4-6
7 Search strategy	Full strategies in supplementary.	Supplementary File
8 Selection process	Methods for selecting studies.	p.4-6
9 Data collection process	Methods for data extraction.	p.4-6
10a Data items	List outcomes.	p.4-6
10b Other variables	List additional variables.	p.4-6
11 Risk of bias	Methods used.	p.6
12 Effect measures	Specify effect measures.	Not applicable

13a Synthesis methods	Eligibility for synthesis.	p. 4-6
13b Data prep	Handling missing data.	Not applicable
13c Tabulation	Presentation methods.	p.4-6
13d Synthesis	Model/methods.	p.4-6
13e Heterogeneity	Exploration methods.	Not applicable
13f Sensitivity analyses	Conducted analyses.	Not applicable
14 Reporting bias	Assessment methods.	p.6
15 Certainty assessment	Evidence certainty.	Not applicable
16a Study selection results	Search results summary.	Figure 1
16b Excluded studies	Cited exclusions.	p.4-6
17 Study characteristics	Characteristics table.	Supplementary
18 Risk of bias in studies	Assessments.	p.6
19 Results of individual studies	Summary data.	p.6-8
20a Synthesis summary	Characteristics summary.	p.6-8
20b Statistical results	If meta-analysis.	Not applicable
20c Heterogeneity causes	Investigations.	Not applicable
20d Sensitivity results	Sensitivity analyses.	Not applicable
21 Reporting biases	Bias assessments.	Not applicable (no meta-analysis conducted due to study heterogeneity)
22 Certainty of evidence	Certainty assessments.	Not applicable

23a Interpretation	General interpretation.	p.8-11
23b Evidence limitations	Limitations.	p.11
23c Review process limitations	Process limits.	p.11
23d Implications	Practice/future research.	p.11
24a Registration	Registration info.	Not registered
24b Protocol access	Protocol details.	No protocol prepared
24c Protocol amendments	Amendments.	Not applicable
25 Support	Financial/non-financial support.	p.13
26 Competing interests	Declare conflicts.	p.14
27 Data availability	Data/material availability.	p. 13-14 Available in the supplementary materials
