# Supplementary material for: Residual Microcalcifications After Neoadjuvant Chemotherapy: Implications for Surgical Decision-Making—A Systematic Review
Source: J Clin Med. 2026 Jan 7;15(2):451. doi: 10.3390/jcm15020451 (PMC12842292; doi:10.3390/jcm15020451)
Supplement: Supplementary file 1 [file jcm-15-00451-s001.zip › Supplementary_Table S1_revised.pdf]

Supplementary Table S1. Summary of studies evaluating residual microcalcifications after neoadjuvant chemotherapy

First Author (Year)	Country	Study Design	Sample Size (N)	Patient Population	Primary Imaging Modality	Pathologic correlation/Outcome	Key Findings
Spring et al. [5]	Multinational	Systematic review and meta-analysis	9,330	Breast cancer patients receiving NAC across subtypes	Not applicable	pCR and survival outcomes	pCR strongly improves survival; strongest in HER2+ and TNBC
An et al .[8]	Korea	Retrospective cohort	29	Women with locally advanced breast cancer (stage II or III) who received NAC	Mammography and MRI	accuracy comparison of MG vs MRI	Residual microcalcifications were malignant in 55.2% of cases; MRI showed moderate agreement
Zhu et al.[9]	China	Retrospective cohort	127	Breast cancer patients with BI-RADS 4-5 microcalcifications who underwent NAC (2010-2020)	Mammography and MRI	accuracy of MG vs MRI	MRI more accurate than MG for residual tumor size evaluation
Li et al.[10]	China	Prospective observational analysis	187 + 48 (control)	Large operable or locally advanced breast cancer patients from Shanghai Cancer Center	Mammography	Patterns of calcifications before/after NACT; efficacy evaluation; surgical decision-making	calcification changes not related to tumor response
Basik et al.[11]	Multicenter (US/Canada)	Phase 2 prospective nonrandomized clinical trial	101 (evaluable)	Operable invasive ductal carcinoma patients with cCR and rCR after NAC	Trimodality imaging (mammography, ultrasound, MRI)	NPV and sensitivity of tumor-bed biopsy	NPV varied by subtype
Goldberg et al.[12]	Israel	Prospective multicenter observational	92	Patients achieving pCR following NAC ± trastuzumab (2011-2013)	Mammography, MRI	Complete eradication of invasive and non-invasive cancer; persistence of microcalcifications	DCIS can be completely eradicated by NAC

Adrada et al.[13]	USA	Retrospective cohort	106	Women with locally advanced breast cancer who underwent NAC (2004-2008)	Mammography	Correlation between extent of microcalcifications and residual disease	Extent of calcifications on mammography after NAC does not correlate with extent of residual disease ; ER+ patients had higher proportion of residual malignant calcifications
Ploumen et al.[14]	Netherlands	Systematic review and meta-analysis	4,987 (31 studies total, 17 for meta-analysis)	Patients with invasive breast cancer with DCIS component treated with NAC	Mammography, breast MRI, contrast-enhanced mammography	Imaging findings of DCIS pre- and post-NAC; evaluation of DCIS response	Calcifications associated with DCIS persist even after complete response
Kim et al.[15]	Korea	Retrospective cohort	370	Invasive breast carcinoma patients who underwent NAC (2015-2018)	Mammography and MRI	Changes in microcalcifications extent/patterns; correlation with pathologic response; oncologic outcomes	No significant differences in microcalcification changes based on pathologic response
Allotey et al.[16]	UK	Retrospective cohort	42	Invasive breast carcinoma patients who underwent NAC (2017-2021)	Mammography	Relationship between pCR and residual microcalcifications	No significant association between pCR and residual microcalcifications
Tasoulis et al.[17]	UK (multicenter)	Multicenter pooled analysis	166	Women ≥18 years with invasive breast cancer treated with NAC, at least partial response	Image-guided biopsy (VAB, core-cut)	Diagnostic accuracy of post-NAC image-guided biopsy	Overall FNR 18.7%; in optimized subgroup (≤2cm, ≥6 VAB samples), FNR 3.2%, NPV 97.4%
Kuerer et al.[18]	USA (7 centers)	Multicentre single-arm phase 2 trial	50	Women ≥40 years with unicentric cT1-2N0-1M0 triple-negative or HER2-positive breast cancer	Image-guided VACB	Biopsy-confirmed ipsilateral breast tumor recurrence rate	VACB identified pCR in 62% of patients; no ipsilateral breast tumor recurrences

Bi et al.[22]	China	Original research study	104	Adult women with histologically confirmed invasive breast carcinoma treated at Shandong Cancer Hospital (2010-2018)	MRI with 3D reconstruction	Modified shrinkage classification modes to guide breast-conserving surgery after NAC	Modified shrinkage modes identified 67.3% cases suitable for BCS
Feliciano et al.[23]	USA	Retrospective cohort	90	Breast cancer patients who underwent NAC (2009-2015) with calcifications within tumor bed	Mammography and breast MRI	Relationship between mammographic calcifications and MRI after NAC; impact on surgical management	no significant correlation between changes in calcifications and MRI enhancement
Golan et al.[24]	Israel	Retrospective cohort	54	Women with pathologically proven breast cancer who underwent NAC (2008-2014)	Mammography (primary), ultrasound, MRI	Correlation between microcalcification changes and tumor response	Patients with decreased calcifications had higher pCR rates
Lee et al.[25]	Korea	Retrospective cohort	144	Locally advanced breast cancer patients with diffuse microcalcifications who received NAC (2011-2017)	Mammography, ultrasonography, MRI	pCR rate, tumor responses to NAC, locoregional recurrence rates	residual cancers found in 80% despite high pCR estimates; complete removal of residual microcalcifications recommended
Kubota et al.[26]	Japan	Consecutive patient analysis	42	Female breast cancer patients candidates for breast conservation treatment with preoperative NAC (2000-2005)	Mammography, ultrasonography, contrast-enhanced MRI	Diagnostic accuracy of imaging modalities in detecting intraductal spread following NAC; predictive ability for residual cancer	Calcification on mammography prior to NAC correlated with intraductal spread on pathological study
Mazouni et al.[27]	USA	Retrospective cohort	2,302	Breast cancer patients treated with NAC (1980-2004)	Not explicitly stated	Overall survival, disease-free survival, local recurrence-free survival	residual DCIS does not adversely affect survival

Qi et al.[28]	China	Retrospective cohort	409	Women with breast carcinoma treated with breast-conserving surgery	Mammography and breast ultrasonography	Local relapse-free survival, disease-free survival, and overall survival	Patients with calcification had significantly increased risk of local recurrence
Sella et al.[29]	Israel	Retrospective analysis	114 (62 without calcifications, 52 with calcifications)	Patients with locally advanced breast cancer who underwent NAC followed by surgery (2011-2018)	MRI (primary), mammography	MRI prediction of response to NAC in tumors with vs without calcifications	residual mammographic calcifications inadequate predictors of residual disease
Thompson et al.[30]	Brazil	Retrospective cohort	115	Post-NAC women with radiologic complete response on MRI (2010-2016)	Pre-treatment MRI and post-treatment mammography	correlation between mammographic findings and residual tumor	residual calcifications on mammography related to higher odds of residual malignancy
Yim et al.[31]	Korea	Retrospective cohort	80	Female patients with locally advanced breast cancer showing radiologic complete response on MR imaging after NAC	Contrast-enhanced MR imaging, mammography, breast ultrasound	Correlation between changes in mammographic microcalcifications and tumor response grade; comparison of calcification extent with lesion extent after NAC	Changes in calcifications showed limited correlation with residual tumor extent, indicating that calcification dynamics alone are insufficient to guide surgical extent.
Choi et al.[32]	Korea	Retrospective cohort	209	Female patients with locally advanced breast cancer showing radiologic complete response on MR imaging after NAC	Contrast-enhanced MR imaging, mammography, breast ultrasound	Radiologic and clinicopathologic factors contributing to radiologic-pathologic discordance (false-negative results)	calcifications in mammography, multifocal multicentric lesions, and non-mass enhancement associated with false-negative findings

Weiss et al.[33]	USA	Retrospective cohort	136	Breast cancer patients treated with NAC, with/without mammographic calcifications	Mammography and MRI	Correlation between calcification extent and pathological tumor size after NAC	Calcification size poorly correlated with pathology; MRI more accurate; calcifications may persist or increase despite tumor response

Abbreviations: NAC, neoadjuvant chemotherapy; NACT, neoadjuvant chemotherapy; NST, neoadjuvant systemic therapy; NAT, neoadjuvant therapy; MRI, magnetic resonance imaging; pCR, pathologic complete response; DCIS, ductal carcinoma in situ; HR, hormone receptor; HER2, human epidermal growth factor receptor 2; TNBC, triple-negative breast cancer; VAB, vacuum-assisted biopsy; VACB, vacuum-assisted core biopsy; FNR, false-negative rate; NPV, negative predictive value; rCR, radiologic complete response; cCR, clinical complete response; ICC, intraclass correlation coefficient; CCC, concordance correlation coefficient; BI-RADS, Breast Imaging Reporting and Data System; RECIST, Response Evaluation Criteria in Solid Tumors; ER, estrogen receptor; PR, progesterone receptor; DFS, disease-free survival; OS, overall survival; IDC, invasive ductal carcinoma; BCS, breast-conserving surgery; RR, relative risk; TTR, time to recurrence; TILs, tumor-infiltrating lymphocytes; ROC, receiver-operating-characteristic.

Notes: This table summarizes the key characteristics of 24 studies investigating mammographic microcalcifications and their relationship to treatment response in breast cancer patients undergoing neoadjuvant therapy. Studies span from 2007 to 2025 and include various designs from retrospective analyses to prospective clinical trials and systematic reviews. Sample sizes range from 29 to 4,987 patients across different study types. The studies represent research from multiple countries including Korea, USA, China, Israel, UK, Brazil, Netherlands, and multicenter international collaborations.
