# Supplementary material for: Residual Microcalcifications After Neoadjuvant Chemotherapy: Implications for Surgical Decision-Making—A Systematic Review
Source: J Clin Med. 2026 Jan 7;15(2):451. doi: 10.3390/jcm15020451 (PMC12842292; doi:10.3390/jcm15020451)
Supplement: Supplementary file 1 [file jcm-15-00451-s001.zip › Supplementary_Table_S2_Search_Strategy.pdf]

Supplementary Table S2. Full Search Strategies Used for Each Database

Detailed, reproducible search strategies for each electronic database searched in this systematic review. Boolean operators, controlled vocabulary, and free-text keywords were applied as shown below.

Database	Search Strategy	Date Range	Notes
PubMed	("breast neoplasms"[MeSH Terms]) AND ("calcification" OR "microcalcification") AND ("neoadjuvant chemotherapy" OR "preoperative systemic therapy" OR "NAC") AND ("residual disease" OR "pathologic complete response" OR "pCR")	January 2000 – May 2025	Filters: Humans, English
Embase	('breast tumor'/exp OR 'breast cancer') AND ('calcification'/exp OR 'microcalcification') AND ('neoadjuvant chemotherapy'/exp OR 'preoperative systemic therapy') AND ('residual disease' OR 'pathologic complete response')	January 2000 – May 2025	Filters: Article, English
Cochrane Library	("breast cancer" OR "breast neoplasm") AND ("calcification" OR "microcalcification")	January 2000 – May 2025	Review & Clinical Trial filters applied

	AND ("neoadjuvant chemotherapy" OR "preoperative therapy")		
Google Scholar	"breast cancer" AND "microcalcification" AND "neoadjuvant chemotherapy" AND "residual disease"	January 2000 – May 2025	First 200 results screened
Scopus	TITLE-ABS-KEY("breast cancer" AND "microcalcification" AND "neoadjuvant chemotherapy" AND "residual disease")	January 2000 – May 2025	English only

All searches were last updated in May 2025. Reference lists of relevant articles were also manually reviewed to ensure completeness.
