# Supplementary material for: Residual Microcalcifications After Neoadjuvant Chemotherapy: Implications for Surgical Decision-Making—A Systematic Review
Source: J Clin Med. 2026 Jan 7;15(2):451. doi: 10.3390/jcm15020451 (PMC12842292; doi:10.3390/jcm15020451)
Supplement: Supplementary file 1 [file jcm-15-00451-s001.zip › Supplementary_Table_S3_Study_Heterogeneity.pdf]

Supplementary Table S3. Study Heterogeneity Summary (Key Studies Only)

This table summarizes key representative studies illustrating heterogeneity in neoadjuvant chemotherapy (NAC) regimens, imaging modalities, calcification classification, and pathologic complete response (pCR) rates among the included literature. It selectively presents the most frequently cited and methodologically influential studies (n=10) to highlight the spectrum of study designs and definitions. A comprehensive dataset including all 24 eligible studies is available in Supplementary Table 1 (Study Characteristics).

Study (Year)	Study Design	Sample Size (n)	NAC Regimen	Imaging Modality	Calcification Classification	% pCR	Main Pathologic Correlation
Zhu et al.[9]	Retrospective	127	Anthracycline + Taxane	MG + MRI	Residual vs Cleared	44	MRI ICC 0.77 vs MG 0.10 – MRI superior in predicting residual tumor.
Li et al.[10]	Retrospective	187 + 48 (control)	Anthracycline + Taxane	MG ± MRI	Dynamic change (↓ ↑ stable)	40	Calcification change pattern not predictive of residual malignancy.
Basik et al.[11]	Prospective Multicenter Trial	101	Mixed NAC ± HER2 therapy	MG + US + MRI + biopsy	Radiologic complete response	Variable	NPV 78.3% overall; 90% in HER2+ subgroup.
Adrada et al.[13]	Retrospective	106	Anthracycline + Taxane	MG ± MRI	Benign/DCIS/Invasive	39	41% benign, 28% DCIS, 31% invasive.
Ploumen et al.[14]	Systematic Review + Meta-analysis	>5000	Various regimens	MG ± MRI	Benign/DCIS/Invasive	30–60	29–60% DCIS; 38–62% benign pathology.
Kim EY et al.[15]	Retrospective cohort	370	Various NAC regimens	Mammography	Residual microcalcifications patterns	Lower pCR in prese	Residual calcifications linked to worse DFS; extensive calcification associated with recurrence

						nce of calcifications	
Tasoulis et al.[17]	Prospective	166	Mixed NAC regimens	MG + MRI + biopsy	Radiologic vs Pathologic CR	46	Image-guided biopsy improves predictive accuracy post-NAC.
Lee et al.[25]	Retrospective	144	Anthracycline + Taxane	MG ± MRI	Benign/DCIS/Invasive	42	Calcifications associated mainly with DCIS, not invasive disease.
Kubota et al.[26]	Retrospective	42	Anthracycline-based	MG	Benign/DCIS/Invasive	35	Microcalcifications often correspond to DCIS; imaging–pathology discordance noted.
Yim et al.[31]	Retrospective	80	Anthracycline + Taxane ± HER2 therapy	MG ± MRI	Benign/DCIS/Invasive	45	Residual calcifications not associated with DFS/OS after pCR.

Caption: Summary of key representative studies illustrating heterogeneity in NAC regimen, imaging modality, calcification classification, and pathologic complete response (pCR) rate.

Abbreviations: MG = mammography; MRI = magnetic resonance imaging; US = ultrasound; CEM = contrast-enhanced mammography; DCIS = ductal carcinoma in situ; DFS = disease-free survival; OS = overall survival; NPV = negative predictive value; CR = complete response.
